# Use of trolamine to prevent and treat acute radiation dermatitis: a systematic review and meta-analysis

**DOI:** 10.1590/1518-8345.2035.2929

**Published:** 2018-05-07

**Authors:** Amanda Gomes de Menêses, Paula Elaine Diniz dos Reis, Eliete Neves Silva Guerra, Graziela De Luca Canto, Elaine Barros Ferreira

**Affiliations:** 1 Undergraduate student in Nursing, Departamento de Enfermagem, Universidade de Brasília, Brasília, DF, Brazil.; 2 PhD, Adjunct Professor, Departamento de Enfermagem, Universidade de Brasília, Brasília, DF, Brazil.; 3 PhD, Adjunct Professor, Departamento de Odontologia, Universidade de Brasília, Brasília, DF, Brazil.; 4 PhD, Adjunct Professor, Departamento de Odontologia, Universidade Federal de Santa Catarina, Florianópolis, SC, Brazil.; 5 Doctoral student, Universidade de Brasília, Brasília, DF, Brazil.

**Keywords:** Review, Radiodermatitis, Skin Care, Radiotherapy, Nursing

## Abstract

**Objective:**

to evaluate the effects of trolamine in the prevention or treatment of radiation dermatitis.

**Method:**

systematic review and meta-analysis. Detailed individual search strategies for Cinahl, Cochrane Library Central, LILACS, PubMed, and Web of Science were developed in January 2016. A manual search was also performed to find additional references. A grey literature search was executed by using Google Scholar. Two researchers independently read the titles and abstracts from every cross-reference. The risk of bias of the included studies was analyzed by the Cochrane Collaboration Risk of Bias Tool. The quality of evidence and grading of strength of recommendations was assessed using Grades of Recommendation, Assessment, Development and Evaluation (GRADE).

**Results:**

seven controlled clinical trials were identified. The controls used were calendula, placebo, institutional preference / usual care, Aquaphor^®^, RadiaCare™, and Lipiderm™. The studies were pooled using frequency of events and risk ratio with 95% confidence intervals, in subgroups according to radiation dermatitis graduation.

**Conclusion:**

based on the studies included in this review, trolamine cannot be considered as a standardized product to prevent or treat radiation dermatitis in patients with breast and head and neck cancer.

## Introduction

The most common effect of radiotherapy is radiation dermatitis, which has greater impact in patients with head and neck and breast cancer^1^. About 80 to 90% of these patients treated by radiotherapy experience radiation dermatitis during treatment^2^ ^-^ ^3^.

The skin is an organ with high radiosensitivity and susceptible to damage by radiotherapy due to rapid cell proliferation and maturation. The epidermis loses a percentage of its basal cell exposure beginning at the first fractionated dose of radiotherapy, and the repeated exposure of the subsequent fractions leads to continuous cell destruction, which avoids tissue repair^4^.

Although the skin damage starts after the first exposure to radiation, the clinical signs are often present from the second week of radiotherapy. They are characterized by mild erythema, which can develop to dry or moist desquamation, and ulcerations in some cases^5^ ^-^ ^6^.

Acute skin reactions generate local discomfort, itching and varied degrees of pain that impact the quality of life of patients and affect the therapeutic efficacy and the planning of radiotherapy, considering that severe intensity lesions can cause interruption of treatment^1^ ^,^ ^7^.

Trolamine has been indicated to prevent and treat radiation dermatitis but, to the best of our knowledge, there is no systematic review that evaluated trolamine as a potential topical product to manage skin reactions due to radiotherapy.

## Background

Skin reactions may be intensified, according to the treatment plan received, a full high dose, fractional high dose, and the extension of the irradiated area. Chemotherapy and patient related factors, such as age, skin color, smoking habits and obesity also aggravate the skin reactions^6^ ^,^ ^8^.

Topical products are commonly used as alternatives to manage skin reactions due to radiotherapy, although there is insufficient evidence regarding skin care products for the prevention or treatment of radiation dermatitis^6^.

Topical application of emulsions containing trolamine has bee used in clinical practice for more than three decades in Europe and in the United States for the management of radiation dermatitis. Trolamine has the capacity to heal through the recruitment of macrophages to the wound, promoting the growth of granulation tissue^9^. Trolamine emulsion is a compound with properties similar to nonsteroidal anti-inflammatory agents and has been considered as a safe and tolerable topical intervention, with low potential to develop contact dermatitis. Trolamine promotes skin hydration and reduces discomfort and pain, which contribute to the non-interruption of treatment^9^.

The evidence and clinical observations demonstrate the advantages and disadvantages between trolamine and other topical products, including steroidal creams, non-steroidal anti-inflammatory compounds, and antihistamines^1^ ^,^ ^10^.

The aim of this study is to systematically review the literature about the evidence of trolamine compared to other topical products in the prevention and treatment of acute radiation dermatitis in cancer patients.

## Method

### Protocol and registration

The reporting of this systematic review adhered to the Preferred Reporting Items for Systematic Reviews and Meta-Analyses PRISMA Checklist^11^. The systematic review protocol was registered at the International Prospective Register of Systematic Reviews (PROSPERO), registration number CRD42016032805^12^.

### Eligibility criteria

Only original prospective studies in which the objective was to investigate the effects of the use of trolamine as the only active ingredient (without associations) to prevent and treat acute radiation dermatitis compared to other topical products in cancer patients undergoing radiotherapy were eligible. Studies published in Portuguese, English, Spanish, and French were included. There were no restrictions to the year of publication. The age of the participants, sex, previous or concurrent therapies, health status or dosage of treatment was not restricted either.

Studies were excluded for the following reasons: 1. cobalt therapy; 2. studies that compared interventions to chronic radiation dermatitis only; 3. trolamine associated with others compounds; 4. trolamine compared with non-topical products; 5. study design: reviews, letters, conference abstracts, personal opinions, book chapter, retrospective study, descriptive study, case reports or case series.

### Information sources and search strategy

Studies were identified using a search strategy adapted for each electronic database, with the aid of a health sciences librarian: CINAHL EBSCO, Cochrane Central Register of Controlled Trials (CENTRAL), LILACS, PubMed, and Web of Science. The hand search was performed on the reference lists from the selected articles for any additional references that might have been missed in the electronic search. In addition, a grey literature search was performed using Google Scholar.

We used the following search terms to search PubMed and adapted the strategy for the other databases: (“biafine” OR “triethanolamine” OR “trolamine” OR “trolamine emulsion” OR “emulsion containing trolamine”) AND (“radiodermatitis” OR “dermatitis” OR “radiation dermatitis” OR “radio-dermatitis” OR “skin damage” OR “skin toxicity” OR “skin reaction” OR “skin injuries” OR “radiation reaction” OR “radio-epithelitis” OR “acute skin toxicity” OR “acute skin reaction” OR “acute dermatitis” OR “acute radiodermatitis” OR “acute cutaneous toxicity” OR “acute radiation dermatitis” OR “acute radiation reactions” OR “acute radiation-induced skin reactions” OR “radiation-induced acute skin” OR “radiation induced skin injuries” OR “radiation-induced skin reaction” OR “radiation induced dermatitis” OR “radio-induced damage” OR “radiotherapy-induced skin reactions” OR “radiation skin reactions” OR “radiation-induced skin injuries”).

After obtaining all references, duplicates were excluded by using appropriate software (EndNoteBasic^®^, Thomson Reuters, USA). All the electronic database searches were undertaken on January 18^th^, 2016.

### Study selection

For the phase of screening and data extraction, ©Covidence (Web-based systematic review tool designed to facilitate the process) was used.

**Figure 1:**
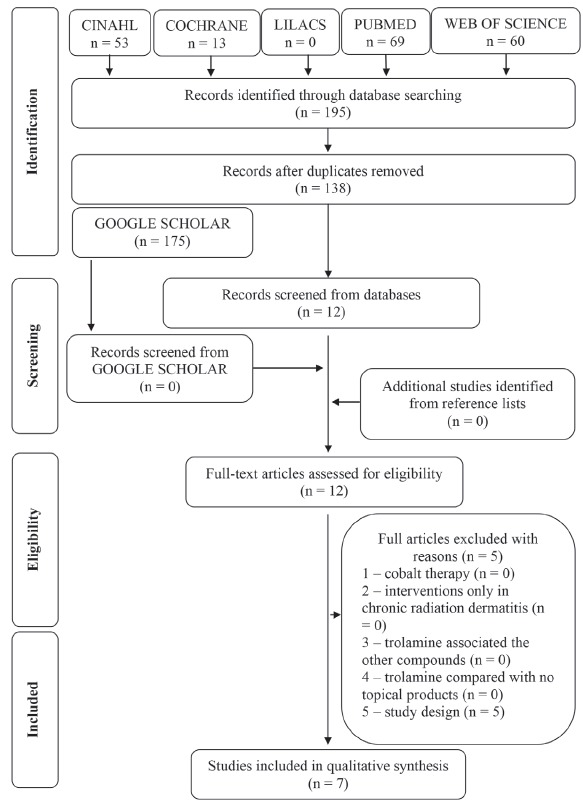
Flow diagram of literature search and selection process. Brasília, DF, Brazil, 2016

The study selection was conducted in two phases. In phase 1, two investigators (A.G.M. and E.B.F.) independently screened the titles and abstracts of potentially relevant studies and selected articles that appeared to meet the inclusion criteria based on their abstracts. In phase 2, the same reviewers independently read the full-text of all selected articles and excluded studies that did not meet the inclusion criteria. Any disagreements, either in the first or second phases, were resolved by discussion and mutual agreement between the two reviewers. In case a consensus could not be reached, a third author (P.E.D.R.) was involved to make a final decision. Studies that were excluded after full-text assessment and the reasons for their exclusion are listed in Figure 1.

### Data collection process and items

Two investigators (A.G.M. and E.B.F.) independently collected the data from the selected articles: study characteristics (author(s), year of publication, setting, objectives, methods), population characteristics (sample size, age, irradiated area), intervention characteristics (groups, follow-up period, primary outcomes, radiation dermatitis criteria and statistical analysis), and outcome characteristics (main results). The third author (P.E.D.R.) crosschecked all the retrieved information to make a final decision. If the required data were not complete, attempts were made to contact the authors to retrieve any pertinent missing information.

### Risk of bias in individual studies

To assess the risk of bias of the included randomized controlled trials (RCT), the Cochrane Collaboration Risk of Bias Tool^13^ was applied, including judgments about the sequence generation, allocation concealment, blinding of participants, personnel and outcome assessors, incomplete outcome data, selective reporting, and other sources of bias. The risk of bias was assessed as low, high or unclear. Two investigators performed this process independently (A.G.M. and E.B.F.). Disagreements between the 2 reviewers were resolved by a third investigator (P.E.D.R.).

### Summary measures

The primary outcome was the development of different grades of radiation dermatitis or the reduction of the intensity/degree of reaction. Further measures considered in this review were risk ratio (RR) or risk differences for dichotomous outcomes.

### Synthesis of results

The overall data combination of the included studies was performed by a descriptive synthesis. Statistical pooling of data using meta-analysis was planned whenever trials were considered combinable and relatively homogeneous in relation to design, interventions and outcomes. Heterogeneity within studies was evaluated either by considering clinical (differences about participants, type of controls and results), methodological (design and risk of bias) and statistical (effect of studies) characteristics or by using the I^2^ statistical test. A value from 0 to 40% was considered of not important consistency, between 30 and 60% moderate heterogeneity, whereas 50 to 90% was considered to represent substantial heterogeneity^13^.

The Cochrane Collaboration´s Review Manager^®^5 (RevMan 5) was used to summarize the results by means of the Mantel-Haenszel model. The results were presented with 95% confidence intervals (95% CI).

### Risk of bias across studies

The quality of evidence and grading of the strength of recommendations was assessed using the Grades of Recommendation, Assessment, Development and Evaluation (GRADE)^14^ ^-^ ^15^. The criteria for this assessment were study design, risk of bias, inconsistency, indirectness, imprecision and other considerations. The quality of evidence must be characterized as high, moderate, low, or very low^15^.

No funnel plot was constructed to assess the possibility of publication bias because there were few trials per subgroups of meta-analysis.

## Results

### Study Selection

**Figure 2:**
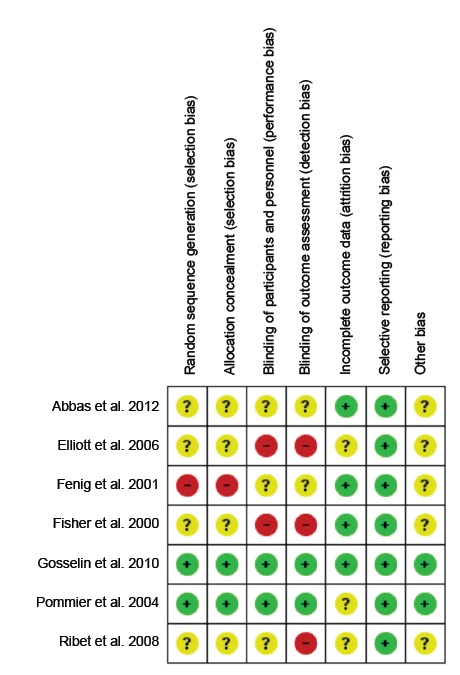
Risk of bias assessment for individual studies. Brasília, DF, Brazil, 2016.

In phase 1 of study selection, 195 citations were identified across five electronic databases. After the duplicated articles were removed, 138 citations remained. No references from grey literature were added. A thorough screening of the titles and abstracts was completed and 126 references were excluded. A manual search from the reference lists of the identified studies yielded no additional studies. Thus, 12 articles remained for a full-text screening (phase 2). This process led to the exclusion of five studies (Figure 1). In total, seven articles^16^ ^-^ ^22^ were selected for data extraction and qualitative synthesis (Figure 2). Figure 1 (flow chart) details the process of identification, inclusion and exclusion of studies with reasons.

### Study characteristics

The studies were published in English^16^ ^-^ ^19^ ^,^ ^21^ ^-^ ^22^ and French^20^, from 2000 to 2012.

Two studies included patients who also underwent concurrent chemotherapy^19^ ^,^ ^22^. Radical radiotherapy has been reported in five studies^16^ ^-^ ^18^ ^,^ ^20^ ^-^ ^21^. The use of tamoxifen has been described in only one study, among those including patients with breast cancer^17^.

Two studies^19^ ^,^ ^22^ included only head and neck cancer patients, and four studies^16^ ^-^ ^18^ ^,^ ^21^ included only breast cancer patients in the sample. Only one^20^ of the selected studies included a heterogeneous sample of patients with different cancer types and irradiated areas: breast and head and neck cancer.

All studies evaluated trolamine as an intervention to prevent radiation dermatitis and only one evaluated trolamine as treatment^19^. The topical controls were usual care/institution routine^16^ ^,^ ^19^ ^,^ ^22^, calendula^18^, water thermal gel^20^, placebo, Aquaphor^®^, RadiaCare™^21^, Lipiderm and no intervention^17^.

Table 1 summarizes the descriptive characteristics of the studies.

**Table 1:** Summary of descriptive characteristics of included articles (n=7). Brasília, DF, Brazil, 2016

Study characteristics		Population characteristics		Intervention characteristics				Outcome characteristics	
Year, Country	Objective	Total *n* Irradiated Area	Age Mean (years)	Intervention (*n*)	Control (*n*)	Follow-Up (months)	Primary outcomes	RD* Criteria	Main Results
2012^(^ ^22^ ^)^ Egypt	To compare trolamine with usual care for patients with head and neck cancer undergoing radiotherapy with concurrent chemotherapy	30 Head and neck	54.5	Trolamine emulsion (15)	Usual care (15)	16	Development of mild reaction (grades 1 and 2), and higher-grade RD*	RTOG^†^ Acute Radiation Toxicity Criteria	Grade 1-2 TA^‡^: 80% (12/15) CA^§^: 46.6% (7/15) *P*< 0.01 Grade 3 TA^‡^: 20% (3) CA^§^: 53.4% (8) *P*<0.01 Grade 4: none
2006^(^ ^19^ ^)^ Canada	To compare trolamine emulsion, as a prophylactic agent and as an interventional agent, with declared institutional preference in reducing the incidence of higher-grade RD*	494 Head and neck	59.0	Trolamine emulsion Prevention (163) Treatment (172)	Institutional preference (159)	19	Reduction of grade 2 or higher RD*	NCI/CTC^||^ version 2.0 ONS^¶^ - toxicity scoring system	Grade 0** TA^‡^: 3% (5/163) CA^§^: 1% (2/159) Grade 1 TA^‡^: 18% (30/163) CA^§^: 20% (31/159) Grade 2 TA^‡^: 54% (88/163) CA^§^: 57% (90/159) Grade 3 TA^‡^: 21% (35/163) CA^§^: 20% (31/159) Grade 4 TA^‡^: 3% (5/163) CA^§^: 3% (5/159) *P* = 0.82
2001^(^ ^17^ ^)^ Israel	To evaluate the effectiveness of Biafine and Lipiderm in preventing RD*	75 Breast	69	Biafine (25)	Lipiderm (24) Control (25)	-	Incidence of RD*	RTOG^†^	Grade 3-4 reaction^††^ TA^‡^: 25% (6/25) Lipiderm: 23% (5/24) Control: 25% (6/25) *P* = 0.98
2000^(^ ^16^ ^)^ United States of America	To compare Biafine to best supportive care in preventing RD*	140 Breast	61	Trolamine (66)	Best supportive care (74)	4	Prevention or reduction of RD* - Time to develop grade 2 or high skin toxicity	RTOG^†^	Grade 0 TA^‡^: 9% (6/66) CA^§^: 7% (5/74) Grade 1 TA^‡^: 50% (33/66) CA^§^: 58% (43/74) Grade 2 TA^‡^: 41% (27/66) CA^§^: 32% (24/74) Grade 3 TA^‡^: 0% (0/66) CA^§^: 3% (2/74)
2010^(^ ^21^ ^)^ United States of America	To evaluate three commonly used skin care products for women receiving whole-breast radiotherapy against a placebo	208 Breast	Placebo 55.8 Aquaphor^*®*^ 54.8 Biafine^*®*^ RE 56 RadiaCare^™^ 55.6	Trolamine (Biafine^*®*^ ) (53)	Placebo (49) Aquaphor^*®*^ (53) RadiaCare^™^ (53)	48	Prevention or reduction of RD*	RTOG^†^	Grade 2 to 4^‡‡^ TA^‡^: 90% (47.7/53) Placebo: 80% (39.2/49) Aquaphor^*®:*^ 80% (42.4/53) RadiaCare^™^ 72% (38.16/53)
2004^(^ ^18^ ^)^ France	To assess the effectiveness of calendula for the prevention of acute RD* of grade 2 or higher during postoperative radiotherapy for breast cancer, compared with trolamine	254 Breast	Calendula 56.5 Trolamine 55.1	Trolamine (128)	Calendula (126)	20	Occurrence of acute RD* of grade 2 or higher	RTOG^†^	Grade 2 to 3 TA^‡^: 63% (95% CI^§§^, 59 to 68) CA^§^: 41% (95% CI^§§^, 37 to 46) *P* < 0.001 Grade 4: none
2008^(^ ^20^ ^)^ France	To evaluate the efficacy and tolerance Avène Thermal Spring Water anti burning gel versus trolamine cream in the prevention of RD*	69 Head and neck Breast	57.9	Trolamine cream (34)	Avène Thermal Spring Water anti burning gel (35)	-	Time to onset of the first signs of RD*	National Cancer Institute	Grade 0 TA^‡^: 24.1% (7/29) CA^§^: 23.3% (7/30) Grade 1 TA^‡^: 34.5% (10/29) CA^§^: 46.7% (14/30) Grade 2 TA^‡^: 34.5% (10/29) CA^§^: 26.7% (8/30) *P* = 0.347

*RD: Radiation Dermatitis; †RTOG: Radiation Therapy Oncology Group; ‡TA: Trolamine Arm; §CA: Control Arm; ||NCI/CTC: National Cancer Institute/Common Toxicity Criteria; ¶ONS: Oncology Nursing Society; **Prevention group; ††Nurse’s impression; ‡‡Data calculated by review authors; §§CI: Confidence Interval.

### Risk of bias within studies

The risk of bias was analyzed individually in all studies included. One randomized clinical trial was graded as having a low risk of bias in the six domains assessed^21^ (Figure 2). Four studies^16^ ^,^ ^19^ ^-^ ^20^ ^,^ ^22^ exhibited an unclear risk of selection bias due to the poor description of the randomization strategy. One of the studies^17^ had a high risk of bias due to randomization description of the inclusion of participants in the intervention groups consecutively. The domain “selective reporting” showed predominantly low risk of bias in the evaluation of the studies (100%).

Four studies were classified as *high risk of bias* because they contained one or more compromised domains^16^ ^-^ ^17^ ^,^ ^19^ ^-^ ^20^. Two studies were classified as *uncertain risk of bias* ^18^ ^,^ ^22^. One of them received positive bias ratings, with low risk of bias in 91% of the evaluated domains^18^. Only one study presented *low risk of bias* in all domains evaluated^21^, allowing us to ascribe the results of the study as of increased reliability.

### Results of individual studies

The studies used trolamine to prevent or treat radiation dermatitis and reported different results for all 7 articles. Characteristics and results of the included studies are listed in Table 1.

### Synthesis of results

Regarding the rating scales, five studies used exclusively the RTOG scale (71.4%)^16^ ^-^ ^18^ ^,^ ^21^ ^-^ ^22^, one of them used only NCI-CTC (14,1%)^20^, and one study used both NCI-CTC and ONS scales to assess the skin reactions of their patients^19^.

**Figure 3:**
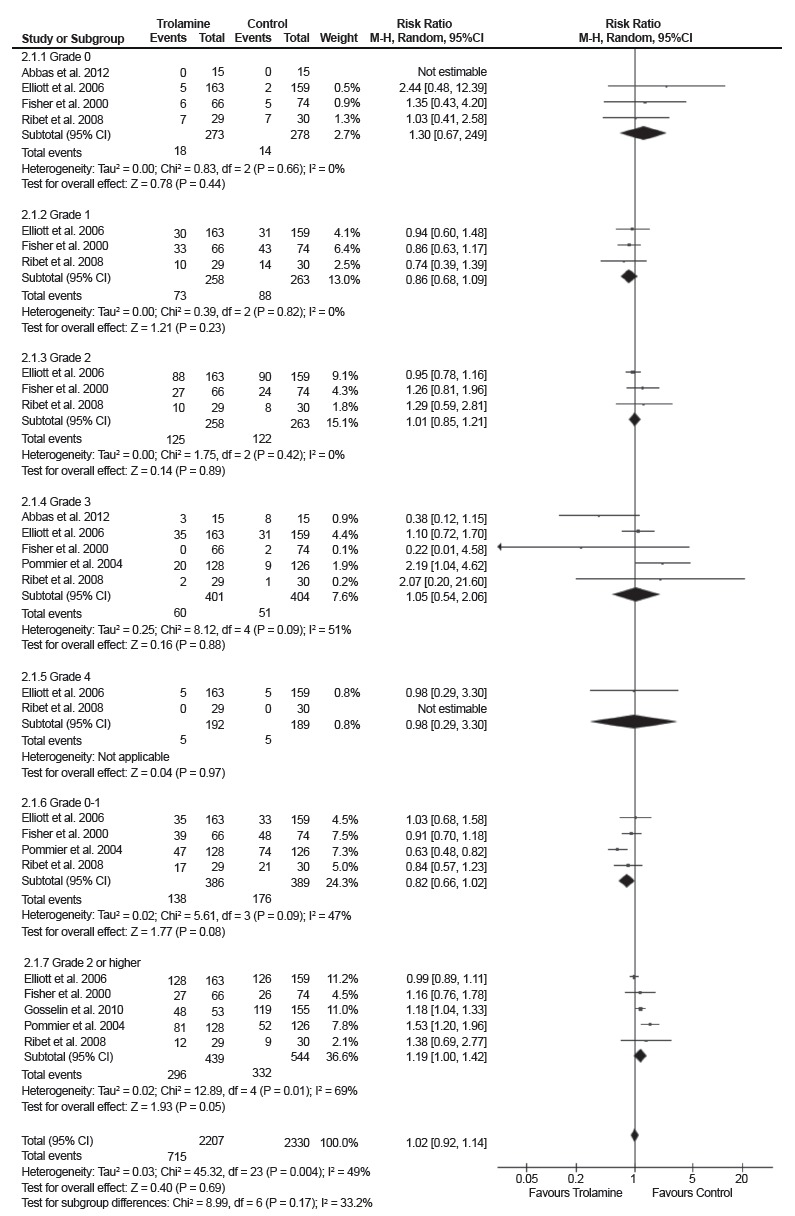
Forest plot of trolamine vs. controls according to the degree of radiation dermatitis

The studies were grouped into subgroups according to the graduation of radiation dermatitis^16^ ^,^ ^18^ ^-^ ^22^. Overall, the results of this random-effect meta-analysis demonstrate that there is no difference between the use of trolamine and evaluated controls to prevent radiation dermatitis (RR 1.02, 95% CI: 0.92 - 1.14. I^2^ = 49%) (Figure 3).

### Risk of bias across studies

**Figure 4:**
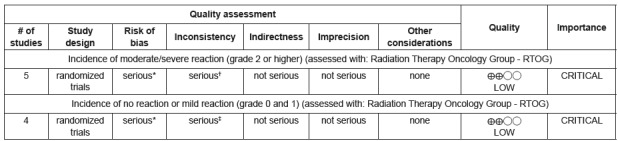
GRADE assessment. Brasília, DF, Brazil, 2016

The quality of the evidence from the outcomes evaluated by the GRADE system was assessed as very low (Figure 4), suggesting very low confidence in the estimated effect based on the outcomes assessed. It means that the true effect is likely to be substantially different from the estimate of effect. The important limitations in the studies and inconsistency were the main factors responsible for the low quality of the evidence from the studies evaluated.

*Two studies had no blinded sample and indicate that the absence of blinding can entail bias. The random sequence generation of three studies is unclear; †I2=69%; ‡I2=47%.

## Discussion

In this review, seven studies evaluating trolamine to prevent or treat radiation dermatitis were included. In four studies^17^ ^-^ ^19^ ^,^ ^21^, no benefits were shown for the use of trolamine to prevent radiation dermatitis and, in two studies^16^ ^,^ ^20^ there was no difference to prevent radiation dermatitis between trolamine and evaluated controls. Only one study^22^ showed satisfactory use of trolamine in the prevention of radiation dermatitis, but its results showed benefit only to prevent grade 3 radiation dermatitis.

Trolamine has been considered because of its good tolerability and its ability to moisturize skin and reduce local discomfort. However, it has not been proven that trolamine is a topical skin radioprotective agent^9^. Some controls presented greater or similar efficacy when compared to trolamine^16^ ^-^ ^21^. According to the meta-analysis, there is no difference between trolamine and controls to prevent radiation dermatitis^16^ ^,^ ^18^ ^-^ ^22^.

The skin moisture and the skin reactions from the radiotherapy could be influenced by the number of intervention applications along the day. Some studies instructed the patients to apply the intervention three times a day^16^ ^,^ ^19^ ^,^ ^22^ or twice daily^17^ ^,^ ^21^ or five times a day^20^. Only one study^18^ allowed patients to apply the intervention twice a day or more according to the frequence of radiation dermatitis and pain. None of this studies described a relation between the frequence of intervention and control applications and the skin moisture. One of the studies^17^ asked patients to start the product application ten days before the onset of radiotherapy, but no contribution was added to prevent radiation dermatitis.

The product quantity in each application was not measured by the studies, except in one of the studies^18^ in which the mean total number of tubes was 1.62 times more used in the trolamine group than in the calendula group.

Patients considered trolamine use more satisfactory than controls when compared to calendula^18^ and Aquaphor^R^ and RadiaCare^R(^ ^21^.

Some studies have shown that chemotherapy and tamoxifen increased the intensity of skin reactions in patients undergoing radiotherapy^23^ ^-^ ^26^. Two studies used chemoradiotherapy^19^ ^,^ ^22^ and, in one study, tamoxifen was used concomitantly with radiotherapy in breast cancer patients^17^, but these studies did not report significant differences in the skin reactions between the groups using trolamine or controls.

Only one study evaluated the efficacy of trolamine to treat radiation dermatitis, and considered no efficacy of trolamine in head and neck cancer patients^19^. It is important that other studies evaluate trolamine to treat grade 1 and grade 2 radiation dermatitis, because these grades require products with moisturizing and anti-inflammatory action. One of the studies^22^ considered that trolamine prevents grade 3 of radiation dermatitis in head and neck cancer patients, but this conclusion is only based on those patients who did not develop grade 3 of radiation dermatitis. Moreover, the non-development of maximum grades of radiation dermatitis depends on extrinsic (total dose, fractionation, radiation energy, volume of treated regions, treatment duration, boost aplication, and treatment site) and intrinsic factors (age, comorbid conditions, skin phototype, and genetic predisposition)^27^.

## Conclusion

Based on the studies included in this review, trolamine cannot be considered as a standardized product to prevent or treat radiation dermatitis in patients with breast and head and neck cancer. Further well-structured blinded studies using trolamine as a treatment are required to evaluated the moisturizing and anti-inflammatory action.
